# Antiviral Functional Foods and Exercise Lifestyle Prevention of Coronavirus

**DOI:** 10.3390/nu12092633

**Published:** 2020-08-28

**Authors:** Ahmad Alkhatib

**Affiliations:** School of Health and Life sciences, Teesside University, Tees Valley, Middlesbrough TS1 3BX, UK; drahmadalkhatib@gmail.com; Tel.: +44-164-273-8239

**Keywords:** functional food, lifestyle prevention, exercise, COVID-19, viral infection, immune system

## Abstract

Novel coronavirus (COVID-19) is causing global mortality and lockdown burdens. A compromised immune system is a known risk factor for all viral influenza infections. Functional foods optimize the immune system capacity to prevent and control pathogenic viral infections, while physical activity augments such protective benefits. Exercise enhances innate and adaptive immune systems through acute, transient, and long-term adaptations to physical activity in a dose-response relationship. Functional foods prevention of non-communicable disease can be translated into protecting against respiratory viral infections and COVID-19. Functional foods and nutraceuticals within popular diets contain immune-boosting nutraceuticals, polyphenols, terpenoids, flavonoids, alkaloids, sterols, pigments, unsaturated fatty-acids, micronutrient vitamins and minerals, including vitamin A, B6, B12, C, D, E, and folate, and trace elements, including zinc, iron, selenium, magnesium, and copper. Foods with antiviral properties include fruits, vegetables, fermented foods and probiotics, olive oil, fish, nuts and seeds, herbs, roots, fungi, amino acids, peptides, and cyclotides. Regular moderate exercise may contribute to reduce viral risk and enhance sleep quality during quarantine, in combination with appropriate dietary habits and functional foods. Lifestyle and appropriate nutrition with functional compounds may offer further antiviral approaches for public health.

## 1. Introduction

Viral infections are responsible for significant global morbidity and mortality rates across the world, and viral outbreaks such as novel coronavirus (COVID-19) [1]. Reports from the World Health Organization (WHO) estimate 3–5 million hospitalized cases of seasonal influenza severe illness, resulting in 290,000–650,000 annual deaths [2]. Currently, COVID-19 is causing a health crisis across the world. Limiting the spread of infections in the short and medium terms involves a number of preventative public health practices including regular hand washing, covering coughs, lockdown, and social distancing measures. Vaccines have been implemented for preventing and controlling several viruses over the past century and have also been used for preventing common influenza [3,4]. However, influenza vaccine development takes a significant amount of time [5], which necessitates alternative complementary remedies for COVID-19. Furthermore, antiviral medication treatments face continuous challenges in terms of drug dose and selection and intervention phase, especially during acute respiratory infections [6]. 

Lifestyle approaches could play an essential antiviral long-term preventative role. The antiviral role of nutrition and exercise as the two lifestyle prevention pillars has received little research attention. In particular, how the antiviral immunological defence capacity could be enhanced using functional foods, nutraceuticals, and physical activity behaviors, whether such behaviors are alone or combined. Functional foods and nutraceuticals can be safe and cost-effective strategies to enhance the immune system and provide protection from pathogenic viral infections. For example, optimal intake of selected micronutrients has been highlighted in controlling the impact of virulent strain infections, including lower and upper respiratory tract infections, through optimizing a well-functioning immune system [7]. On the other hand, the role of physical activity and exercise in enhancing the immune system is well established [8]. Nutrition and lifestyle modifications may not be definitive measures to absolutely prevent persons from contracting COVID-19 when exposed. However, they could help as an adjuvant therapy to reduce the risk through enhanced immunity. This review presents key evidence on how functional foods and lifestyle approaches, including physical activity, effective for cardiometabolic disease prevention outcomes [9], can also optimize the immune system response to viral infection, especially respiratory tract infections and COVID-19. The review also makes specific and practical evidence-based recommendations for the use of antiviral functional foods and lifestyle approaches.

## 2. Antiviral Role of Exercise Immunology

In terms of physical activity prevention of chronic disease morbidity and mortality risks, a dose response relationship is well established for non-communicable disease (NCD) prevention [10]. Exercise induces cardiovascular, respiratory, and metabolic adaptations, which result in higher maximal oxygen uptake ($\dot{V}$ O_2max_), carbon dioxide production, minute ventilation, breathing frequency, stroke volume, and cardiac output [11]. Improvement in whole-body cardiometabolic and respiratory functions boosts the immune system defence through several acute and long-term mechanisms, which have been well highlighted recently [12,13]. However, less is reported about how such exercise-dependent mechanisms protect against communicable diseases (CDs), especially viral infections such as influenza and the recent COVID-19 outbreak.

Exercise impacts all immune cells within both innate and adaptive immune systems, particularly elevating the activity of natural killer (NK) cells, neutrophils, and macrophages following moderate exercise (less than 60% of $\dot{V}$ O_2max_) [8]. For example, acute aerobic exercise (running, cycling) have been shown to increase monocyte number [14]. Monocytes play an integral antiviral role, and it has been shown that in a variety of influenza A viruses (including the circulating swine-origin virus, similar to COVID-type viruses), exercise induces monocytes to differentiate within 18 hours into CD16(-)CD83(+) mature dendritic cells with enhanced capacity to activate T-cells [15]. Long-term moderate exercise training increases the expression of T-helper (Th) cells and associated Th balances [16]. Enhanced T-cell proliferation was particularly found following prolonged moderate exercise training in high-risk populations such as postmenopausal women cancer survivors and the very old. An increase in T-cell proliferation (by 218 per dpm × 106 cells) has been found in post-menopausal breast cancer survivors who exercised for 15–30 min for 15 weeks at 50% and 70% of $\dot{V}$ O_2max_ [17], while increased CD4+ T-cells were also found in older adults aged 80 years, walking 30 km per day [18]. Those high-risk groups have been found to be particularly vulnerable to viral infections and more serious symptoms as reflected by recent governmental advice regarding COVID-19 [19,20]. Exploring the exercise role in protecting and controlling COVID-19 and other novel viral infections is essential.

Severe exercise intensity, whether acute or chronic, can be counter-productive in terms of the susceptibility to infections, since it is linked with higher upper respiratory tract infection rates among elite endurance athletes [21,22]. Mucosal immunology antibodies such as salivary immunoglobulin A (IgA), and immunoglobin M (IgM) concentrations have been shown to decline immediately after a bout of intense exercise in elite swimmers, but usually recover within 24 h [22]. However, modulating the intensity and duration of exercise can optimize the immune system response outcomes acutely and chronically [8,23]. Higher exercise intensities (above 70% $\dot{V}$ O_2max_) and supramaximal exercise (100% or above) induce a transient oxidative stress and muscle damage response of oxidative stress, cell integrity, and homeostasis biomarkers, especially during the first 24 hour post exercise [24,25]. Both young and older adults have shown an increase in recombinant interleukin-2 (rIL-2) stimulation of NK cells immediately (15 min) following an acute intense bout of maximal cycling exercise [26]. When followed for 12 days, it was found that neutrophil mobilization was concurrent with enzyme efflux, particularly those related to cell damage such as creatine kinase (CK) and antioxidative capacity such as superoxide dismutase (SOD) [27]. Acute repeated exercise bouts have also been implicated in the removal and regeneration of aged immune cells, especially cell senescent naïve, memory CD4+ and CD8+ T-lymphocytes, and an elevated apoptotic lymphocyte in peripheral blood [28].

The immunological transient response includes a temporal stress (e.g., disturbance of cell homeostasis and oxidative stress) induced by an acute exercise challenge, and may play a role in long-term enhanced immune system, especially when exercise is repeated chronically (i.e., exercise training). These entropic exercise-induced effects on the immune system may act as a natural vaccine against viral infections such as COVID-19. In fact, eccentric exercise has already been demonstrated to act as an adjuvant to influenza vaccination in humans [29]. The trial randomized 60 healthy men and women who performed upper body eccentric exercise (deltoid and biceps brachii muscles) 6 hours prior to receiving influenza vaccination, and were monitored for antibody titers up to 20 weeks. The results showed that interferon-gamma responses were enhanced by exercise in men, whereas antibody titres were enhanced in women, which were concurrent with improved arm circumference (i.e., physical outcome benefit). The interferon–gamma response was positively associated with the percentage increase in arm circumference. The study suggested that eccentric exercise of the muscle at the site of vaccine administration could act as a behavioral adjuvant to vaccination. Therefore, exercise immunological benefits alone or as an adjuvant antiviral treatment should be further investigated for preventing and controlling COVID-19.

The immune function response to exercise is influenced by several factors including nutritional status, body weight, hygiene, and mental health. The immune function is known to be superior in highly conditioned versus sedentary individuals. Sedentary lifestyle and insufficient physical activity levels induce several physiological impairments, which reflect reduced cardiovascular and respiratory capacity, obesity, and associated cardiometabolic chronic diseases [30,31]. Consequences of sedentary lifestyle and physical inactivity include a compromised immune system due to manifestation of systemic inflammation, oxidative stress, and associated immunosuppressive mechanisms [10,31]. Prevalence of sedentary behavior and low physical activity levels have been reported in those with obesity, diabetes, and underlying insulin resistance and oxidative stress, and have been linked with increased susceptibility to contracting viral infections, including pandemic influenzas such as H1N1 and COVID-19 [32]. Conversely, higher physical activity and fitness levels in adults are associated with an optimized immunity indicated by reduced white blood cell count, C-reactive protein (CRP), interleukins (IL-6, and IL-18), tumor necrosis factor alpha (TNF-α), and other inflammatory biomarkers [33]. Therefore, any physical activity or exercise dose is considered beneficial compared to being sedentary, especially during and after COVID-19-related lockdown, social distancing, or quarantine measures introduced in several countries.

### Exercise Recommendations for COVID-19 Prevention

Moderate exercise intensity is recommended, especially during and after a social distancing lockdown, which requires an avoidance of severe intensities. A practical method of achieving moderate exercise intensity is using 40–70% of maximum heart rate (HR_max_ = 220-age). Exercising at home, especially to perform resistance type activities using own body weight, and to interrupt sedentary behavior by reducing sitting times are particularly recommended for older and high-risk individuals with chronic conditions such as diabetes [31,34]. Exercise at home is also suited for the avoidance of the airborne coronavirus, especially during quarantine, and may include strengthening, balance and control, stretching, or a combination of these (walking, lifting and carrying, lunges, stair climbing, stand-to-sit and sit-to-stand using house items, squats, sit-ups, yoga) [35]. A volume increase in weekly exercise is recommended under the COVID-19 quarantine from 150 min to 200–400 min aerobic exercise distributed across 5–7 days, with at least 2–3 resistance sessions, to compensate for the decreased mobility during lockdown [36]. This results in achieving an increase in $\dot{V}$ O_2max_ as a practical aim. Enhanced $\dot{V}$ O_2max_ is particularly important for those who are considered at high-risk of COVID-19 such as those who are overweight or those with obesity, insulin resistance, and diabetes, who typically have chronic low-grade inflammation characterized by increased levels of several pro-inflammatory cytokines and the inflammasome, and who are predisposed to greater risks for infection along with more adverse outcomes [37]. It is recommended that exercise is performed as part of a multicomponent personalized lifestyle approach (personalized nutrition, exercise intensities, technology, behavior, mental wellbeing) especially for high-risk individuals such as those with diabetes [38].

## 3. Importance of Functional Foods in Preventing Communicable Disease and COVID-19

Functional foods naturally possess active ingredients or “nutraceuticals” that are associated with disease preventative health benefits are now widely accepted for the prevention and management of major NCDs, especially those characterized by inflammatory and oxidative stress disorders such as diabetes and cardiovascular disease [9,39]. However, less is known about the role of functional foods in communicable diseases (CDs), especially on the immune system defence against viral infections such as COVID-19. A variety of fruits, vegetables, oily fish, olive oil, nuts, legumes are all considered functional foods based on their natural contents of nutraceuticals, including polyphenols, terpenoids, flavonoids, alkaloids, sterols, pigments, and unsaturated fatty acids [9,40]. Polyphenol-rich herbs, especially coffee, differently fermented teas (green, black)and yerba maté, have also shown to have various effectiveness on metabolic and microvascular activities, cholesterol and fasting glucose lowering, anti-inflammation and anti-oxidation in high-risk populations [9,41]. Bioactive peptides, naturally present in food proteins or formulated as nutraceuticals based on their molecular weight, amino acid chain length, or peptide composition, have also been postulated to elicit versatile physiological responses associated with immunological, antimicrobial, cardiovascular, gastrointestinal, neurological, and other hormonal activities of the human system [42]. Such functional food benefits can be translated to protect against viral infections and COVID-19.

Viral infections are characterized by a compromised immune function and deficient micronutrient stores, particularly vitamins, including vitamins A, B6, B12, C, D, E, and folate, and trace elements, including zinc, iron, selenium, magnesium, and copper [7]. Evidence already supports an efficient function of the immune system through consuming those various nutraceuticals within a variety of functional foods including essential fatty acids, linoleic acids, essential amino acids, and the aforementioned vitamins and minerals, especially where forms of immunity may be affected by deficiencies in one or more of these nutraceuticals [43,44]. Adequate dietary intake, and supplementation of such functional foods, contribute to maintaining optimal levels in the human body, which enhances several aspects of the immune system [7,45], and provides an important antiviral prevention of COVID-19 [46]. Conversely, less robust immune responses have been shown to be the primary risk factor for COVID-19 [47], which makes it timely to describe the protective role of functional food component benefits in the context of preventing COVID-19 and seasonal infections.

In terms of jointly addressing NCD and CD prevention within high-risk populations, investigating the functional foods effects on CDs including COVID-19 is particularly important. Higher infection and mortality rates related to COVID-19 have been documented among older adults and patients with obesity, cardiac diseases, hypertension, or diabetes [48]. For example, COVID-19 statistics in England showed that almost a third (31.3%) of COVID-19-related mortalities had type-2 diabetes [49], while there was a two-fold increase (86%) in requiring mechanical ventilation among COVID-19 infected obese individuals compared with (47%) of infected healthy weight individuals [50]. The prevalence of NCDs, especially diabetes amongst high-risk groups, is becoming a matter of emerging importance, and diabetes is now considered a risk factor for the progression and prognosis of COVID-19 [51,52]. Therefore, optimal “immune-enhancing” functional foods combined with behavioral lifestyle approaches (especially exercise) could provide an optimal prevention of the double burdens of NCD and CD multimorbidity.

Various dietary patterns contain functional food components that have been promoted in the past for NCD prevention, especially the vegetarian diet, the Nordic diet, or the Mediterranean diet (MD), and its combination with other lifestyle approaches [9,39,53,54]. Common functional foods within those diets include plant-based fruit and vegetables such as olive oil and tree nuts, seeds, fish, dairy products, and herbs, teas, and fermented products, which contain key nutraceuticals with disease protective anti-inflammatory and anti-oxidation properties [9,54,55]. Established health protective functional components include monounsaturated fatty acids (MUFA) such as oleic acid in olive oil, omega-3 polyunsaturated fatty acids (e.g., alpha-linolenic acid) found in tree nuts such as walnuts, eicosapentaenoic acid (EPA), and docosahexaenoic acid (DHA) found in oily fish, high amounts of polyphenol flavonoids and antioxidants found in fruit and vegetables, and high amounts of fiber found mainly in cereal and whole-grain foods [54]. Consuming those functional foods, and their components vary across geographical global regions [9,41,54,56,57,58], but what is agreed on is their cardiometabolic protective benefits of reducing major NCDs and mortality risks [9,53,59]. The challenge is to translate such functional effects towards enhancing and protecting the immune system and its antiviral defence response into the prevention of emerging CDs such as COVID-19.

## 4. Functional Foods Mechanisms of Optimizing the Immune System Antiviral Defence

Enhancing the antiviral immune defence can benefit from the functional food intake of a considerable variety of plant, animal, and fungi species, consumed across different diets and cultural practices including traditional herbal medicine such as teas, roots, mushrooms, and fermented plants and leaves; MD components such as olive-based products, oily fish, seeds, fruits, and vegetables; popular beverages such as coffee; and protein-rich foods such as chicken extract and soybean peptides. The majority of such foods contain naturally occurring vitamins and minerals (e.g., vitamins C, D, B_6_, B_12_, A, E, and minerals of zinc, copper, iron, and selenium), and other phenolic compounds that are immunoprotective particularly through antioxidation and anti-inflammation properties [41,43]. Other foods such as oily fish omega-3 fatty acids contain monounsaturated fatty acids such as omega-3 fatty acids (EPA and DHA) in oily fish, which can be enzymatically converted to specialized pro-resolving mediators (SPMs) known as resolvins, protectins, and maresins, which are molecules that support inflammatory resolution and healing of infected sites including the respiratory tract, which could prevent acute lung injury [7]. Fermented food products (e.g., yoghurt, pickles, fermented fruits, vegetables, plant, and drinks) contain probiotics, and have also been shown to enhance gut bacteria profile and gut–lung axis-related respiratory fitness [60,61]. A summary of systematic reviews and randomized controlled trials reported reduced incidence and severity of upper and lower respiratory tract infections (odds ratio ∼ 0.8–0.5) by using different probiotics, especially lactobacilli and bifidobacteria [61]. The efficacy of probiotics in reducing COVID-19 infected patients has not yet been established, but the prophylactic benefits for enhancing the immune system are supportive of their long term use [60], especially considering that improving gut microbiota profile has been recently implicated in preventing COVID-19 in older and high-risk individuals with compromised immune systems [62].

Selected food supplements and micronutrient vitamins and trace elements have been reviewed elsewhere in terms of optimizing the immune responses [7,63,64]. Other reviews have highlighted the importance of key vitamins (e.g., vitamin D) for regulating sleep patterns during quarantine or lockdown measures [65,66,67]. Given the promising role of popular functional foods, such as those within the MD including olive oil, and Asian and African herbal teas and fermented foods and popular beverages as part of lifestyle disease prevention [11,40], it is important to contextualize the antiviral mechanisms of such functional foods. Below is a review of popular foods within various dietary patterns, including olive oil nutraceuticals, popular vitamins such as vitamin D, traditional medicinal herbs and roots, and protein peptides for preventing viral infections including COVID-19, especially when they are adopted as part of an active lifestyle.

### 4.1. Potential Antiviral Benefits of Olive Oil Nutraceuticals

Olive oil (OO), and its constituents (leaves and bark), form an important immune-enhancing functional food due to the significant NCD preventative benefits, especially of cardiovascular disease, diabetes, and cancer, which have been reviewed elsewhere [40,68]. OO, especially extra-virgin OO (EVOO) contains monounsaturated fatty acids, and several polyphenols including oleuropein and hydroxytyrosol, which have several antioxidative and anti-inflammatory properties, which can be linked with significant antiviral and antibacterial potential. Oleuropein has shown a potential antiviral activity against respiratory syncytial virus (RSV), a common upper respiratory infection (URI) virus [69]. This effect has been attributed to the antioxidative property of elenolic acid as a main fragment in oleuropein, which has long been shown to have potent antiviral activities against herpes, influenza A and B, and parainfluenza 1, 2, and 3 viruses [70]. Antioxidant capacity of OO was later shown to be independent of the size of the antiviral effect, with oleuropein showing superior antiviral effects compared with other secoiridoid glucosides isolated from ligustrum lucidum [69]. However, antioxidant properties can vary among OO phenolics. A more recent study by Paiva–Martins et al. (2010) [71] compared the capacity of four important OO phenolic compounds, oleuropein, hydroxytyrosol, and the oleuropein aglycones 3,4-dihydroxyphenylethanol-elenolic acid (3,4-DHPEA-EA) and 3,4-ihydroxyphenylethanol-elenolic acid dialdehyde (3,4-DHPEA-EDA) for their protection of red blood cells (RBCs) from oxidative haemolysis induced by the physiological initiator H2O2. The study tested the amount of haemolysis by spectrophotometry, and the compounds were also tested in the presence and absence of the naturally occurring antioxidant ascorbic acid. All compounds were revealed to significantly protect RBCs from oxidative haemolysis induced by H2O2 at 40 and 80 µM, with the order of activity being 3,4-DHPEA-EDA>3,4-DHPEA-EA>hydroxytyrosol=oleuropein. However, at 20, 10, and 5 µM, only 3,4-DHPEA-EDA showed a significant protection against the oxidative injury, suggesting that 3,4-DHPEA-EDA plays an important protective role against reactive oxygen species-induced oxidative injury in RBCs, and this effect is more potent than the one evidenced by hydroxytyrosol or oleuropein. 

The antioxidation protective benefits of OO, especially EVOO, which has a higher phenolic content [40] promotes its role for enhancing the immune system defence against viruses. Hydroxytyrosol antiviral mechanisms were showed through its inactivation effects on influenza A viruses, especially during the virus morphological changes, such as the presence of a viral envelope which is an integral membrane protein involved in several aspects of the virus life cycle including its assembly, budding, and pathogenesis [72]. The mechanisms of which OO nutraceuticals protect against viral infections have often focused on the hydroxytyrosol preventative effects on HIV from entering the host cell and binding the catalytic site of the HIV-1, and its inhibitory effect on both viral entry and integration [73]. Regular intake of olive leaf extracts, rich in polyphenol flavonoids, have been shown to be responsible for a 33% reduction in URI [74]. Such promising antiviral potential was attributed to the following antioxidation actions of oleuropein with dose-dependent inhibition of the copper sulphate-induced oxidation of low-density lipoproteins (LDLs), and induced increase in nitric oxide production in macrophages and functional activity. In another study among high-school athletes who were prone to URI, olive leaf extract supplementation (20 g, containing 100 mg oleuropein) was shown to reduce the duration of infection (28% reduction in sick days) but not the incidence rate [75]. Thus, olive polyphenols (both in OO and leaf), especially oleuropein and hydroxytyrosol, seem to promote antiviral defence and can be an adjacent prevention to control URIs. Exploring OO mechanisms for protecting against novel viruses such as COVID-19, especially its protein viral envelop function and interaction with host cells would also be important.

The benefits of OO intake, especially as part of a balanced diet such as the MD can be further augmented via physical activity, especially strength and resistance type exercise [40]. Such an approach is likely to be an effective prevention of viral infections. In terms of the recommended OO dose, a moderate dose of 20–30 g/day (especially polyphenol-rich EVOO) in combination with other dietary functional foods can be recommended for enhancing the immune system, which is in line with recent NCD prevention recommendations [68].

### 4.2. Vitamin D Antiviral Role

The role of vitamin D in ameliorating the effects of both NCDs and CDs is now well accepted. Vitamin D reduces acute respiratory tract infection, and its deficiency is linked with susceptibility to viral infections, and also with various cancers, diabetes, and CVD [64,66]. Research into the role of vitamin D in preventing COVID-19 and influenza viral infections has gained recent momentum through various reviews and meta-analyses, especially given that individuals who are susceptible to COVID-19 infections are mainly high-risk individuals with various NCDs such as diabetes and CVD, suggesting that vitamin D could be the missing link between NCDs and viral CDs [46,66]. Based on the latter [66], several mechanisms of how adequate vitamin D availability can reduce the risk of viral infections and COVID-19 through the following mechanisms: (a) Lowering viral replication rates through cathelicidins and defensins, and preventing lung injures that lead to pneumonia through its anti-inflammatory cytokines; (b) potential for vitamin D supplementation effectiveness in reducing the risk of influenza especially in deficient individuals; (c) documented reduced risk of COVID-19 during the summer indicated by a lower number of cases in the southern hemisphere, compared with higher number of cases in the winter months when 25-hydroxyvitamin D (25(OH)D) concentrations are lowest; (d) vitamin D deficiency has been found to contribute to acute respiratory distress syndrome; and that case-fatality rates increase with age and with chronic disease comorbidity, both of which are associated with lower 25(OH)D concentration. 

Sleep disorders during COVID-19 quarantine could also be ameliorated through maintaining adequate vitamin D levels within the body. This could mainly be done through sun exposure [76], and to a smaller extent from dietary intake or supplementation [77]. Vitamin D plays an important role in regulating sleep patterns, circadian rhythm, enhancing sleep quality, and indirectly ameliorating sleep apnea [78]. For example, vitamin D receptors and the enzymes that control its activation and degradation have been found in brain regions involved in sleep regulation; and vitamin D is also involved in melatonine production pathways, and can affect restless legs syndrome and obstructive sleep apnea syndrome [67].

The best source of vitamin D is sunshine exposure, but it is abundant in several foods including oily fish, tuna, dairy, and egg yolk. Grant et al. (2020) [46] recommended supplementation in individuals with highest risk of COVID-19 infection or vitamin D deficiency (10,000 IU/d of vitamin D3) to raise 25(OH)D concentrations above 40–60 ng/mL (100–150 nmol/L). For treatment of people who become infected with COVID-19, higher vitamin D3 doses are recommended.

### 4.3. Antiviral Role of Functional Foods in Popular Traditional Medicine

#### 4.3.1. Traditional Herbs and Roots

Traditional antiviral medicinal therapies across different cultures are essentially based on a combination of several functional foods and nutraceuticals with active immunomodulators, polyphenols, anti-inflammatory, and anti-oxidation components. Armeniacae semen (apricot seeds), Cinnamomi Cortex (Chinese cinnamon), Glycyrrhizae radix (liquorice root), and Ephedrae Herba form a Japanese traditional medicine called “Maoto”, which is often administered orally as granules to adults with seasonal influenza [79]. It has been shown to be well tolerated and associated with equivalent clinical and virological efficacy to neuraminidase inhibitors in helping progeny influenza viruses to leave without re-infecting the host cell [80]. Licorice roots which contain the active component Glycyrrhizin, have been shown to inhibit influenza A virus uptake into the cell, and reduced CCID50 by 90% [81]. Licorice and curcumin have recently been reviewed for their postulated antiviral potential [82].

Other common traditional herbal remedies for respiratory viruses that have been supported by scientific evidence include berries’ extracts, Echinacea, Clinacanthus siamensis, Punica granatum (pomegranate), Psidium guajava Linn. (guava tea), Epimedium koreanum Nakai; Scutellaria baicalensis Georgi (Baicalin), and Paeonia lactiflora Pall. (Bai Shao) [79]. Examples of their antiviral role include reduction in viral replication, enhancement of anti-influenza virus IgG and IgA antibody production, improvement of T-cell function (e.g., stimulation of interferon-gamma production by T-cells), neuraminidase inhibitor, virus budding prevention, inhibition of viral RNA and viral protein synthesis, viral haemagglutination, viral binding to and penetration into host cells [79]. Fungi are also commonly used in Asian and Chinese medicine to enhance the immune system. For example, Cordyceps militaris is a mushroom traditionally used for diverse pharmaceutical purposes in East Asia, including China, for enhancing immunity. In a human study 1.5g/day of Cordyceps militaris for 4 weeks enhanced the NK cell activity and lymphocyte proliferation and partially increased Th cytokine secretion [83]. Immune enhancing antiviral mechanisms of traditional medicine especially roots and fungi are important preventing and controlling novel influenza viruses, including COVID-19.

#### 4.3.2. Peptide and Amino Acids

Promising evidence has been shown about the restorative and antioxidative role of traditional medicinal herbs and peptides post trauma or physical challenges, which may be important in lung injury pathology. For example, the Chinese ginseng Rg1 herb has been shown to restore satellite cell depletion following an exercise challenge, through enhancing glutathione (GSH), and GSSG [84]. GSH is considered important in immune modulation, remodeling of the extracellular matrix, apoptosis, and mitochondrial respiration through its gamma-glutamylcysteine synthetase heavy and light subunits oxidant/antioxidant response to phenolic antioxidants, and is considered key to the development of an oxidant/antioxidant imbalance in lung inflammation [85]. In another study it was shown that anserine, beta-alanyl-3-methyl-l-histidine, a dipeptide, replenished the free radical scavenging enzymes SOD (superoxide dismutase) and preserved catalase and GSH cofactors, while preserving cell integrity and homeostasis, together with a haematological increase in red blood cell volume-to-concentration and an attenuated white blood cellelevation following muscular challenge in healthy men [24]. Dietary intake of anserine, carnosine dipeptides, and other animal-based amino acids including taurine, creatine, and 4-hydroxyproline promote the immunological defence of humans against infections by bacteria, fungi, parasites, and viruses (including coronavirus) through enhancing the metabolism and functions of monocytes, macrophages, and other cells of the immune system [86,87]. Plant-based peptides from soybean have also been shown to modulate cellular immune systems (increased lymphocytes and granulocytes number, increased CD11b(+) cells and CD56(+) natural killer cells), regulate neurotransmitters (decreased adrenaline and increased dopamine), and boost brain function [88]. However, fish, meat, and poultry are the primary sources of immunomodulatory peptides and amino acids, and hence they have long been considered functional foods taken to alleviate fatigue, respiratory, and cold symptoms in older individuals, especially in Asia [86,87,89].

#### 4.3.3. Plant Cyclotides

Plant cyclotides are well-studied antivirals, since they can be mimicked for antiviral drug development, given their stable chemical structure [90]. They have been originally extracted from African tea used in traditional African medicine to accelerate childbirth because of their postulated uteroactive antiviral HIV properties [90]. The protective mechanisms of plant cyclotides against infections and pathogens are postulated through preventing malfunctioning of the immune cells by growth-inhibiting growth effects on the human immune system especially on lymphocytes (e.g., T-cells), which can cause an over-reactivity of this defence machinery during infections [91]. Cyclotides can be obtained from various plants including Violaceae and Rubiaceae, but are abundant in several other plant families, especially Cucurbitaceae (e.g., squash, pumpkin, zucchini), Fabaceae (legumes, peas, beans), and Solanaceae (eggplant, tomato, potato, pepper). Therefore, it is likely that consumption of such foods, especially seasonal intake plays a protective role in enhancing the immune system and enhances antiviral defence mechanisms.

#### 4.3.4. Coffee and Caffeine

Coffee, caffeine, and naturally caffeinated beverages are well known to induce various health benefits and prevention of disease. All forms of coffee consumption (differently roast beans, fermented or non-fermented leaves) are common across various cultures across the world for centuries [92,93]. Epidemiological evidence suggests that consuming 2–3 cups of coffee daily is associated with reduced incidence of metabolic diseases which are often concurrent with a compromised immune system such as diabetes [41,94]. Therefore, it is plausible to imply a positive role for caffeine as a useful immunomodulator. Nutraceuticals within coffee have shown different antiviral outcomes, with caffeic acid inhibiting the multiplication of influenza A virus in vitro, whereas caffeine, quinic acid, and chlorogenic acid do not [95]. Caffeic acid has also been shown to have antiviral activity against herpes simplex virus (DNA virus) and polio virus (RNA virus), and to decrease the progeny virus yield (especially within 3 h post-infection) and suppresses the degeneration of the virus-infected cells [95,96]. However, caffeine reported immuno-protective mechanisms from laboratory in vivo and in vitro trials have been equivocal [97,98]. Positive caffeine effects on innate immunity involve suppression of neutrophil and monocyte chemotaxis, and pro-inflammatory cytokines (such as TNF-α) from human blood, but caffeine has also been reported to suppress antibody production and human lymphocyte function as indicated by reduced T-cell proliferation and impaired production of Th1 (IL-2 and interferon--gamma), Th2 (IL-4, IL-5), and Th3 (IL-10) cytokines [99]. Some of the immunomodulatory actions of caffeine have been explained by its inhibitions of cyclic adenosine monophosphate (cAMP)-phosphodiesterase, and consequential increase in intracellular cAMP concentrations [99]. However, recent in vitro evidence suggests that caffeine may suppress endotoxins lipopolysaccharide (LPS)-induced inflammatory responses by regulating nuclear factor NF-κB activation and MAPK phosphorylation [100]. LPS activation of NF-κB triggers mucin transcriptors (e.g., MUC2 gene) and respiratory tract mucus in response to respiratory pathogens including influenza viruses [101]. Caffeine suppression of LPS has also been reported in a recent human study in females with obesity [102]. The latter study also found that caffeine ameliorates the obesity-induced metabolic side-effects following intense exercise lifestyle intervention including elevated LPS, insulin action, glucose homeostasis, and androgen levels. This suggests that caffeine optimizes the metabolic and immunoprotective benefits when combined with other lifestyle components, especially exercise. Future research is needed to determine caffeine antiviral effects for the prevention and management of COVID-19.

## 5. Conclusions and Recommendations

**Figure 1:**
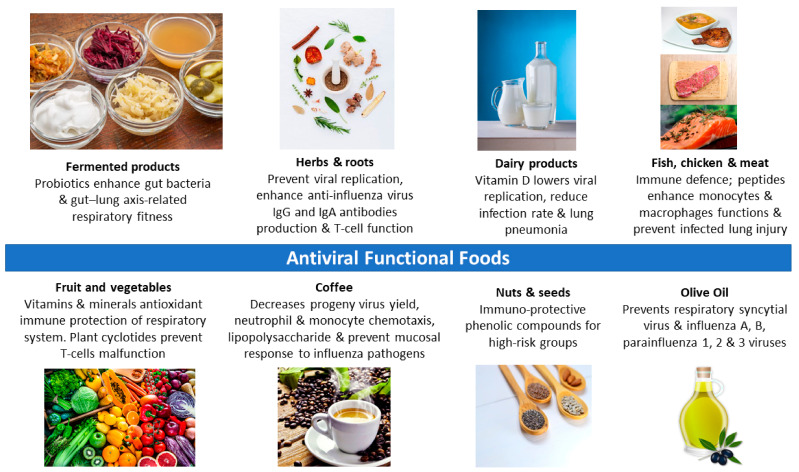
Functional foods and antiviral mechanisms to optimize health.

Exercise and physical activity enhance the immune system and reduce susceptibility to infections, especially respiratory infections including COVID-19. Moderate intensity exercise can be adopted by the large population including high-risk groups with NCDs such as those with diabetes and cardiovascular disease. Functional foods may provide a further effective diverse antiviral approach and could have a joint prevention of both NCDs and CDs among diverse populations. Dietary intake of foods rich in vitamins and minerals can be increased to provide an immune boost, especially in individuals with deficiency in these micronutrients. Increased intake of probiotics, omega-3 from fish, protein peptides from chicken and fish, and olive-based products are also recommended (Table 1, Figure 1). There is no specific model to follow to enhance the immune system against COVID-19. However, the more varied the dietary sources, the better the protection is against all viral infections. Adopting exercise together with an enhanced dietary intake of functional compounds may contribute as a preventative medicine against emerging viral infections.

## Figures and Tables

**Table 1:** Antiviral functional foods, their immune protective nutraceuticals, mechanisms of action, and recommended intake.

Antiviral Functional Foods	Immune-Promoting Nutraceuticals	Key Mechanisms of Action	Antiviral Targeted Recommendations
Fruit & vegetables	Vitamins: C, B2, B6, and B12, folic acid, beta carotene, iron, plant cyclotides	Promote antioxidation and anti-inflammation properties, protect the respiratory system, and reduce risks of infection and re-infection [44]. Cyclotides protect against infections and pathogens by preventing malfunctioning of the immune cells (T-cell lymphocytes), which reduces over-reactivity of this defence machinery during infections [91].	Intake is highly recommended as part of a balanced diet. Complements an active lifestyle, supports circadian rhythm, and sleep quality
Dairy products	Vitamins D, A, & E	Vitamin D reduces the risk of contracting respiratory infections and COVID-19 [46,64]. Lowers viral replication rates through cathelicidins and defensins, and prevents lung injures that lead to pneumonia through anti-inflammatory cytokines [66].	Dietary intake is preferred. Supplements (zinc, selenium, and vitamin D) are recommended in older adults and the most deficient. Enhances sleep quality.
Seeds and nuts	Zinc, selenium, copper, trace minerals	Contain phenolic compounds that are immunoprotective particularly through antioxidative and anti-inflammatory properties in high-risk adults [9].	Supplementation is recommended when dietary intake is low, especially in older and high-risk individuals
Fish & seafood	EPA & DHA Omega-3	Support inflammatory resolution and healing of infected sites including the respiratory tract, which could prevent acute lung injury, mainly through pro-resolving mediators (SPMs) such as resolvins, protectins, and maresins [7].	Increased intake is recommended in high-risk individuals
Protein rich foods (e.g., red meat, chicken, seafood)	Amino acids and peptides: Anserine, carnosine, taurine, creatine, and 4-hydroxyproline, vitamins, iron, copper	Dietary intake of anserine and carnosine promote immunological defence against infections by bacteria, fungi, parasites, and viruses (and coronavirus) through enhanced immune cell functions of monocytes and macrophages [24,86]. Plant peptides (e.g., soybean) increase lymphocytes and granulocytes; enhance natural killer activity [88].	Dietary intake is sufficient, but an increased intake is recommended in high-risk individuals and infected patients. Can be obtained from both animal and plant sources.
Olive based products (olive oil, olive leaves)	Oleuropein, hydroxytyrosol, elenolic acid, vitamin E	Reduced upper respiratory infection attributed to antioxidative property of oleanolic acid in oleuropein, especially influenza A and B, parainfluenza 1, 2, and 3 viruses, and herpes [69].	Dietary intake (20–30 g/day), especially from extra-virgin olive oil, which is high in polyphenol content. Increase benefits with physical activity.
Coffee (coffee leaves, differently fermented)	Caffeic acid, caffeine, polyphenols, chlorogenic acid	Caffeic acid decreases the progeny virus yield (especially within 3 h post-infection) and suppresses the degeneration of the virus-infected cells; caffeine can suppress of neutrophil and monocyte chemotaxis, and pro-inflammatory cytokines (e.g., TNF-α) [96]. It suppresses endotoxins LPS-induced inflammatory responses (regulates NF-κB activation and MAPK phosphorylation) [102], and prevents mucosal response to pathogens infecting the respiratory tract and influenza viruses [101].	Coffee intake (2–3 cups/daily) is recommended and has superior immunological benefit to caffeine supplementation since it is more wholesome (contains both caffeic acid and caffeine).
Roots & fungi, traditional herbs, and medicinal plants	Maoto, licorice roots, cordyceps mushrooms, Chinese mushrooms, ginseng	Herbs and roots prevent viral replication, enhance anti-influenza virus IgG and IgA antibodies production, and improve T-cell function [80]. Glycyrrhizin (in Maoto) helps progeny influenza viruses to leave without re-infecting, inhibits influenza A virus uptake into the cell and reduces CCID50 by 90% [82]. Ginseng and cordyceps have antioxidative (GSH, SOD) and cell senescence angiogenesis properties [84].	Dietary intake is highly recommended. Supplement when dietary intake is low (e.g., cordyceps, 1.5 g/day).
Fermented foods & probiotics	Yoghurt, kaffir, pickles, fermented fruits, vegetables and plants, probiotic drinks	Microbiota especially lactobacilli and bifidobacterial enhance gut bacteria profile and gut–lung axis-related respiratory fitness [61,62].	Dietary intake of fermented foods is recommended

COVID-19, Novel corona virus-19; EPA, Eicosapentaenoic acid; DHA, Docosahexaenoic acid; GSH, Glutathione; SOD, Superoxide dismutase; IgG, Immunoglobulin g; IgA Immunoglobulin A; LPS, Lipopolysaccharides; TNF-α, Tumor necrosis factor-alpha; NF-κB, Nuclear factor-κB; MAPK, Mitogen-activated protein kinase.
